# A de novo missense mutation in *PPP2R5D* alters dopamine pathways and morphology of iPSC-derived midbrain neurons

**DOI:** 10.1093/stmcls/sxae068

**Published:** 2024-10-26

**Authors:** Jasmine L Carter, Julian A N M Halmai, Jennifer J Waldo, Paula A Vij, Maribel Anguiano, Isaac J Villegas, Yu Xin Du, Jan Nolta, Kyle D Fink

**Affiliations:** Center for Interventional Genetics, University of California, Davis, Sacramento, CA 95817, United States; MIND Institute, University of California, Davis, Sacramento, CA 95817, United States; Stem Cell Program and Gene Therapy Center, University of California, Davis, Sacramento, CA 95817, United States; Institute for Regenerative Cures, University of California, Davis, Sacramento, CA 95817, United States; Department of Neurology, University of California Davis Health Systems, Sacramento, CA, United States; Center for Interventional Genetics, University of California, Davis, Sacramento, CA 95817, United States; MIND Institute, University of California, Davis, Sacramento, CA 95817, United States; Stem Cell Program and Gene Therapy Center, University of California, Davis, Sacramento, CA 95817, United States; Institute for Regenerative Cures, University of California, Davis, Sacramento, CA 95817, United States; Department of Neurology, University of California Davis Health Systems, Sacramento, CA, United States; Center for Interventional Genetics, University of California, Davis, Sacramento, CA 95817, United States; MIND Institute, University of California, Davis, Sacramento, CA 95817, United States; Stem Cell Program and Gene Therapy Center, University of California, Davis, Sacramento, CA 95817, United States; Institute for Regenerative Cures, University of California, Davis, Sacramento, CA 95817, United States; Department of Neurology, University of California Davis Health Systems, Sacramento, CA, United States; Center for Interventional Genetics, University of California, Davis, Sacramento, CA 95817, United States; MIND Institute, University of California, Davis, Sacramento, CA 95817, United States; Stem Cell Program and Gene Therapy Center, University of California, Davis, Sacramento, CA 95817, United States; Institute for Regenerative Cures, University of California, Davis, Sacramento, CA 95817, United States; Department of Neurology, University of California Davis Health Systems, Sacramento, CA, United States; Center for Neuroscience, University of California Davis, Sacramento, CA 95817, United States; Center for Interventional Genetics, University of California, Davis, Sacramento, CA 95817, United States; MIND Institute, University of California, Davis, Sacramento, CA 95817, United States; Stem Cell Program and Gene Therapy Center, University of California, Davis, Sacramento, CA 95817, United States; Institute for Regenerative Cures, University of California, Davis, Sacramento, CA 95817, United States; Department of Neurology, University of California Davis Health Systems, Sacramento, CA, United States; Center for Interventional Genetics, University of California, Davis, Sacramento, CA 95817, United States; MIND Institute, University of California, Davis, Sacramento, CA 95817, United States; Stem Cell Program and Gene Therapy Center, University of California, Davis, Sacramento, CA 95817, United States; Institute for Regenerative Cures, University of California, Davis, Sacramento, CA 95817, United States; Department of Neurology, University of California Davis Health Systems, Sacramento, CA, United States; Stem Cell Program and Gene Therapy Center, University of California, Davis, Sacramento, CA 95817, United States; Institute for Regenerative Cures, University of California, Davis, Sacramento, CA 95817, United States; Center for Interventional Genetics, University of California, Davis, Sacramento, CA 95817, United States; MIND Institute, University of California, Davis, Sacramento, CA 95817, United States; Stem Cell Program and Gene Therapy Center, University of California, Davis, Sacramento, CA 95817, United States; Institute for Regenerative Cures, University of California, Davis, Sacramento, CA 95817, United States; Department of Neurology, University of California Davis Health Systems, Sacramento, CA, United States

**Keywords:** NDD, iPSC-derived neuron, midbrain neuron, stem cell, disease modeling, Jordans Syndrome, EOPD, RNA editing and CRISPR/Cas13

## Abstract

Induced pluripotent stem cell (iPSC) models of neurodevelopmental disorders (NDDs) have promoted an understanding of commonalities and differences within or across patient populations by revealing the underlying molecular and cellular mechanisms contributing to disease pathology. Here, we focus on developing a human model for PPP2R5D-related NDD, called Jordan syndrome, which has been linked to Early-Onset Parkinson’s Disease (EOPD). Here we sought to understand the underlying molecular and cellular phenotypes across multiple cell states and neuronal subtypes in order to gain insight into Jordan syndrome pathology. Our work revealed that iPSC-derived midbrain neurons from Jordan syndrome patients display significant differences in dopamine-associated pathways and neuronal architecture. We then evaluated a CRISPR-based approach for editing heterozygous dominant G-to-A mutations at the transcript level in patient-derived neural stem cells. Our findings show that site-directed RNA editing is influenced by sgRNA length and cell type. These studies support the potential for a CRISPR RNA editor system to selectively edit mutant transcripts harboring G-to-A mutations in neural stem cells while providing an alternative editing technology for those suffering from NDDs.

## Graphical Abstract

**Graphical Abstract:**
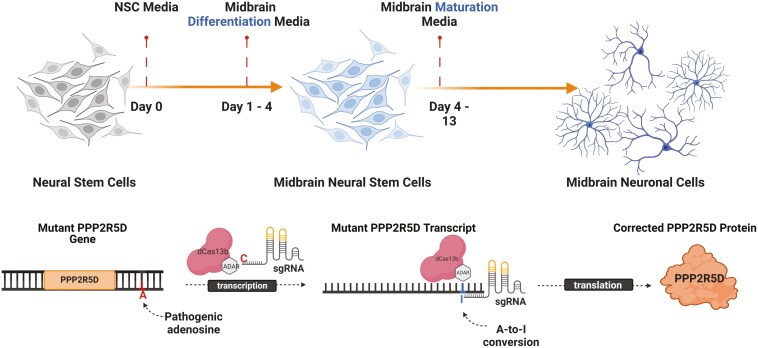

**Significance statement**

This is the first report of iPSC-derived neurons from Jordan's Syndrome, providing an in-depth characterization of the most common mutation. Our findings also link the cell model to reports of EOPD. We also provide evidence and guidelines for performing CRISPR-mediated RNA editing.

## Introduction

Neurodevelopmental disorders (NDD) are a heterogenous class of disorders that impact cognition, adaptive learning, movement, and language development or progression.^1-4^*De novo* heterozygous dominant missense mutations in the protein phosphatase 2 regulatory subunit B’delta (*PPP2R5D)* gene are causative for Jordan syndrome, a rare genetically linked NDD.^5,6^ SFARI gene scoring categorizes *PPP2R5D* as a high-confidence syndromic disorder associated with intellectual disability and autism spectrum disorder.^7^ Clinical data shows *PPP2R5D* mutations specifically contribute to neurological dysfunction such as intellectual disability, seizures, macrocephaly, speech, and motor impairments.^5,6,8,9^ Like many NDD risk genes, variants in *PPP2R5D* can contribute to unique patient-specific clinical phenotypes. A subset of individuals with *PPP2R5D* variants display limited motor coordination and post-mortem brain tissue revealed apparent neuronal loss in the substantia nigra pars compacta. These early-onset Parkinson’s disease (EOPD) phenotypes were responsive to levodopa treatments.^10-12^ Apart from the known role of *PPP2R5D* in dopamine synthesis and neurotransmission, how *PPP2R5D* variants contribute to disease at a molecular and cellular level is unclear.^13-15^

*PPP2R5D* encodes B56δ, a protein that regulates protein phosphatase 2A (PP2A) substrate specificity and catalytic activity at serine/threonine sites.^16^ PP2A is a heterotrimeric complex containing an A scaffolding subunit, B regulatory subunit, and C catalytic subunit. The most frequent pathogenic *PPP2R5D* variants are located in domains of the B56δ subunit that interact with the catalytic subunit to form the substrate recognition and dephosphorylation site. Thus changes in the amino acid charge could result in altered heterotrimeric assembly, substrate binding, and dephosphorylation.^5^ Given the link between *PPP2R5D* variants and NDDs, a strategy for targeting *PPP2R5D* could advance the understanding of the molecular and cellular basis of dysregulation in neurodevelopment. Whether Jordan syndrome results from *PPP2R5D* gain or loss of function is unknown, which supports the developmental of a corrective approach.^5,6,8,17^

The most common *PPP2R5D* variant arises from pathogenic guanosine to adenosine (G-to-A) mutation at the 198 amino acid residue which changes glutamic acid (E) to lysine (K) (E198K). Adenosine deaminase acting on RNA (ADAR) proteins post-transcriptionally modify double-stranded RNA (dsRNA) to catalyze adenosine-to-inosine RNA editing and recognition of inosine as guanosine by translational machinery.^18,19^ ADAR activity has been employed to achieve site-directed RNA editing (SDRE) and catalyze protein recoding, thus creating a unique opportunity to target G-to-A mutations and rescue protein function.^20-22^ With the versatility of CRISPR/Cas orthologs, a CRISPR-based strategy for SDRE can be achieved by fusing the RNA targeting *Prevotella sp. P5-125* Cas13 (*Psp*dCas13b) to the ADAR2 deaminase domain (ADAR2DD).^23^ CRISPR-based SDRE is a novel tool for single nucleotide editing at the transcript level; however, to date, this technology has not been employed to edit endogenous pathogenic G-to-A mutations in patient-specific disease models.

Here, we created an induced pluripotent stem cell (iPSC)-derived neuronal model from E198K patient fibroblasts to understand the underlying molecular and cellular phenotypes of *PPP2R5D* pathology across multiple cell states. The largest set of differentially expressed genes between the E198K and the isogenic line was observed in an early neural stem cell (NSC) state. A midbrain neuronal model revealed expression of canonical midbrain markers, upregulation of genes associated with dopamine secretion in E198K neurons, and morphological differences in neuronal complexity. Finally, we used iPSC-derived NSCs that harbor the endogenous E198K G-to-A mutation to achieve SDRE with *Psp*dCas13b-ADAR2DD. *Psp*dCas13b-ADAR2DD RNA editing of the E198K variant is dependent on sgRNA length and A-C mismatch position. This work confirms that RNA editing in a patient-derived disease model of Jordan syndrome is possible at more than 10% editing frequency based on a transient delivery method.

## STAR methods

### Generation of human iPSC (hiPSCs) and isogenic control from *PPP2R5D* E198K patient

iPSCs were generated from passage 2 patient-derived fibroblasts using the Cytotune 2.0 OKSM Sendai virus (Life Technologies #A34546) on mouse embryonic feeders in Knockout Serum Replacer medium (Life Technologies #10828-028). iPSCs were manually passaged or passaged with collagenase IV for feeder culture. A subset of iPSCs was converted to a feeder-free culture system using StemFlex medium on matrigel, passaged, and frozen using dispase. Undifferentiated human iPSCs were cultured in Corning Matrigel hESC Qualified Matrix (Corning #354277) coated 6-well plates with StemFlex medium kit (GIBCO ThermoFisher #21103049). Cells were routinely cultured at 37 °C in a standard 5% CO_2_ incubator with every other day medium change. hiPSC colonies were monitored and selected for unwanted differentiation. Once the confluency reached 90%, hiPSCs were manually lifted using a cell scraper. Cell aggregates were replated at a ratio of 1:6 wells. An iPSC isogenic line was created with a sgRNA targeting the patient-specific E198K variant in *PPP2R5D* using CRISPR-mediated homology-directed repair, courtesy of Synthego. Sanger sequencing of the E198K and isogenic iPSC lines was conducted to verify the patient variant and correction of the mutation. Additional iPSC lines, the PGP1 series, were generated from a healthy patient-derived parental cell line, PGP1, using CRISPR-mediated homology-directed repair, courtesy of Synthego. E197K, E198K, E200K, and E420K were introduced to the parental line to generate the individual mutant cell line respectively.^24^

### hiPSC differentiation to NSCs

Feeder-free E198K and isogenic hiPSCs were subjected to NSC differentiation over a 12-day period. On day 1, hiPSCs were plated into Aggrewell 800 plates (STEMCELL Technologies #34815) in embryoid body (EB) medium containing Knockout DMEM/F12 (Life Tech # 12660012), 15% Gibco KnockOut Serum Replacement (Life Tech # 10828028), 100X MEM non-essential amino acids (Life Tech #11140050), 100X Glutamax Supplement (Life Tech #35050061), 0.55 mM 2-mercaptoethanol (Life Tech #21985023) and Y-27632 RHO/ROCK pathway inhibitor (STEMCELL Technologies #72304). On day 2, EBs were plated in suspension in low-attachment plates in EB medium supplemented with Noggin (R&D Systems #6057-NG-025) and SB43152 (R&D Systems 1614) over 4 days with media change on day 4. On day 5, EBs were plated onto Corning Matrigel Growth Factor Reduced (GFR) Basement Membrane Matrix (Corning #354230) and subsequently transitioned to neural stem cell medium containing Neurobasal Medium (Life Tech #21103049), GlutaMAX Supplement (100×) (Life Tech #35050061), MEM non-essential amino acids solution (100×) (Life Tech #11140050), penicillin-streptomycin Solution (Corning #30-002-CI), N2 supplement (Life Tech # 17502048) and 10 µg/mL Recombinant human FGF basic/FGF2/bFGF (R&D Systems #233-FB-025), over days 6–12. Rosettes were enzymatically lifted using Accutase (STEMCELL Technologies #7920) and plated on poly-L-ornithine solution (PLO) (Sigma-Aldrich #P4957-50ML) and laminin from Engelbreth-Holm-Swarm murine sarcoma basement membrane (Sigma-Aldrich #L2020-1MG) coated plates supplemented with neural stem cell medium.

### NSC cell culture

NSC lines were maintained on poly-L-ornithine (PLO) and laminin-coated plates in Neurobasal Media (Life Tech #21103049) supplemented with GlutaMAX Supplement (100×) (Life Tech #35050061), MEM non-essential amino acids solution (100×) (Life Tech #11140050), Pen/Strep, Gibco N-2 Supplement (100×) (Life Tech # 17502048), 20 ng/mL Recombinant Human FGF basic/FGF2/bFGF (R&D Systems #233-FB-025), 20 ng/mL Human EGF Recombinant (R&D Systems #236EG200), Serum-free B-27 Supplement (50X) (Life Tech #17504044), 10 mg/mL Insulin Solution Human Recombinant (Sigma-Aldrich #SIAL-I9278-5ML), and D-glucose (Life Tech #A2494001). NSC lines were supplemented with Y-27632 RHO/ROCK pathway inhibitor (STEMCELL Technologies #72304) for 24 hours when passaged. Full media changes were performed every other day and Accutase (STEMCELL Technologies #7920) was used once 95% confluency was reached to lift NSCs for passaging. NSCs were maintained in 75 cm^2^ flasks (Corning #430641U) at 50 000 cells/cm^2^ and plated in 6 well-plates (Corning 3516) at 50 000 cells/cm^2^ for molecular and cellular characterization.

### MTT assay

Across 3 passages, isogenic and E198K NSCs were seeded at 32 000 cells per well in 96 well plates (Corning #3598). Following the manufacturer’s protocol, 24 hours post-seeding NSCs were incubated in 100 µL fresh media supplemented with 10 µL MTT (12 mM) for 2 hours at 37 °C in 5% CO_2_ (Thermofisher #V13154). Following incubation, 85 µL of media with MTT was removed and 50 µL of DMSO was added to each well. NSCs were incubated for 10 minutes at 37 °C in 5% CO_2_ and absorption of the MTT reagent was evaluated on a microplate reader at 540 nm.

### NSC differentiation to neurons

Isogenic and E198K NSCs were plated at a density of 2000 cells/cm^2^ on poly-L-ornithine (PLO) and laminin-coated plates in NeuroCult NS-A Differentiation Kit (STEMCELL Technologies #05752). Full NeuroCult media changes were performed every other day over the course of 23 days. For assessments in midbrain iPSC-derived neurons, Isogenic and E198K NSCs were plated at a density of 50 000 cells/cm^2^ on poly-L-ornithine (PLO) and laminin coated plates in NSC media. Twenty-four-hours post-seeding media was changed to STEMdiff Midbrain Neuron Differentiation Kit (STEMCELL Technologies #100-0038) supplemented with Human Recombinant Sonic hedgehog (Shh) C2411 (STEMCELL Technologies #78 065.2) to differentiate NSCs into midbrain NSCs. Once midbrain NSCs reached 80% confluency, cells were plated at 15 000 cells/cm^2^ on poly-L-ornithine (PLO) and laminin-coated plates to allow for midbrain NSCs to mature into midbrain neurons in STEMdiff Midbrain Neuron Maturation Kit (STEMCELL Technologies #100-0041) over 9 days. All differentiation experiments were performed across 3 independent passages with biological groups of 3-6 (NeuroCult) or groups of 12 (STEMdiff Midbrain).

### Immunocytochemistry

iPSCs, NSCs, midbrain NSCs, and midbrain neurons were fixed in 4% paraformaldehyde diluted in PBS (BioWorld #30450002-1) for 15 minutes at room temperature. The fixative was removed and cells were stored at 4 °C overnight in a wash buffer (ThermoFisher # 00-4954-56). A 0.1% Triton X 100 (Millipore Sigma, #T8787) in phosphate-buffered saline (PBS) solution was used to permeabilize cells for 15 minutes prior to a 1-hour blocking incubation with BSA (BioWorld #22070004) at room temperature. A 3-hour incubation at room temperature in primary antibody (Supplementary Table S1) solution was followed by 3 washes with wash buffer for 5 minutes each. Cells were then incubated for 1 hour in secondary antibody (Supplementary Table S2) and washed 3 times with wash buffer for 5 minutes each. A final nuclear staining with Hoechst was completed for 5 minutes, cells were then stored in a wash buffer for imagining and analysis.

### High-content analysis of neuronal morphology and complexity

iPSC-derived neurons from the NeuroCult and midbrain differentiation experiments were imaged with a CX7 CellInsight HCS Platform. Images were processed using the high-content screening (HSC) neurite outgrowth assay to identify cell bodies, neurites, and branchpoints based on neuronal marker detection and morphology as previously described.^25^

### Quantitative polymerase chain reaction (qPCR)

MIQE guidelines were followed for qPCR experiments.^26^ Cells were washed with PBS and lysed using TriZol Reagent. RNA extractions were performed on Isogenic and E198K iPSCs and NSCs with the Direct-zol RNA Miniprep kit (Zymo Research) and subsequently used for reverse transcription with RevertAid First Strand cDNA Synthesis Kit (Thermofisher, #K1621) with random hexamer primers according to the manufacturer’s guidelines. Twenty nano grams of cDNA per reaction, 5 µM of primers (Supplementary Table S3), and PowerUp SYBR Green Master Mix (Thermo Fisher Scientific) were used for quantitative PCR in biological triplicates and conducted with the StepOne Plus Real-Time PCR system (Thermo Fisher Scientific). Relative expression analysis was calculated as the delta CT between housekeeping (GAPDH) primers and primers amplifying the target gene transcript levels.

### Western blot

Protein was extracted from iPSCs, NSCs, and midbrain NSCs after 4 days in differentiation media using Pierce RIPA Buffer (Thermo Fisher Scientific, Catalog #89901) with Halt Protease and Phosphatase Inhibitor Cocktail (Thermo Fisher Scientific, Catalog #78442) and then quantitated using a Bicinchoninic acid assay protein kit (EMD Millipore, Kit 71285-3). Protein lysate (7.5 µg) was loaded into wells of 4%-20% Stacking TGX Gels (BioRad, Hercules, Catalog #4561096EDU) were electrophoresed for 3 hours at 90 V, then transferred onto prepared Invitrolon PVDF membrane (Thermo Fisher Scientific, Catalog LC2005) and left overnight at 30 V. Membranes were blocked with 10% SEABLOCK (Thermo Fisher Scientific, Catalog #37527) and subsequently incubated for 2 hours with primary antibodies (Supplementary Table S4) in 5% SEA BLOCK in 0.1% TBST. Membranes were then washed with TBST 3 times and incubated with secondary antibodies (Supplementary Table S5) in 5% SEABLOCK in 0.1% TBST for 1 hour. Gels were washed twice in 0.1% TBST and stored in TBS at 4 °C. The Odyssey CLx (LI-COR) was used to image membranes and bands were quantified with Empiria Studio.

### RNA-sequencing

RNA-sequencing was performed on isogenic and E198K iPSCs (*n* = 3), NSCs (*n* = 3), midbrain NSCs (*n* = 4), and midbrain neurons (*n* = 2). Following a PBS rinse, cells were lysed using TriZol Reagent. RNA extractions were performed with the Direct-zol RNA Miniprep kit (Zymo Research). Samples were prepped according to Novogene sample submission guidelines and subsequently used to create strand-specific RNA libraries and sequenced on an Illumina NovaSeq 6000 (PE150). Sequencing reads were de-multiplexed and aligned to the Hg38 reference genome with STAR Universal Aligner version 2.5.3a using the following settings: *Indexed Reference Genome:* Ensembl reference genome and annotation files for Hg38 release 77 were downloaded and compiled into a single file, Genome was indexed using the following arguments ‘STAR –runMode genomeGenerate –runThreadN 12 –genomeDir /STAR INDEX HG38 –genomeFastaFiles GRCh38 r77.all.fa –sjdbGTFfile Homo sapiens.GRCh38.77.gtf –sjdbOverhang 149’; *Sample Read Alignment:* alignment of each sample’s reads was performed with the following arguments: “STAR –runThreadN 24 –genomeDir /STAR INDEX HG38 – outFileNamePrefix /STAR/SampleName –outSAMtype BAM SortedByCoordinate –outWigType bedGraph –quantMode TranscriptomeSAM GeneCounts – readFilesCommand zcat –readFilesIn Sample-R1.fastq.gz Sample-R2.fastq.gz.” Differential expression (DE) analysis was performed with edgeR software in R Studio. Gene count files were combined into a single file and compared to the respective isogenic cell state. DE gene lists from pairwise comparisons were exported into.csv files and utilized for GO term analysis using DAVID. Volcano plots were generated using ggplot2 software in R studio. Heatmaps were generated using pheatmap software in R studio. DEG interaction STRING maps were generated using the STRING-database (STRING-db).

### Cloning of sgRNA

For the cloning of *PPP2R5D* sgRNA, 50 base pair sequences were selected to target the E198K (**A**AA) variant in exon 5. Six sgRNA in total were designed, with the A-C mismatch falling in position 30, 34, 35, 36, 38, or 40. sgRNA were cloned into the *Psp*dCas13b crRNA expression vector (Addgene plasmid #103854) following the previously described protocol.^27^*PPP2R5D* sgRNA of varying length (50-250 base pairs) and fixed A-C mismatch positions as well as *PPP2R5D* sgRNA 190 version 2 with variable A-C mismatch positions were cloned as described above. sgRNA targeting *PPIB*^27^ were cloned as described and used as controls for RNA editing. Sanger sequencing (Genewiz, Inc.) and SnapGene software (from GSL Biotech; available at snapgene.com) were used to confirm sgRNA cloning. Four available *Psp*dCas13b-ADAR2DD constructs were screened: *Psp*dCas13b-ADAR2DD(E488Q) (Addgene plasmid #103849), *Psp*dCas13b-ADAR2DD(E488Q)-delta-984-1090 (Addgene plasmid #103 869), *Psp*dCas13b-ADAR2DD(E488Q/T375G) (Addgene plasmid #103 870), and *Psp*dCas13b-ADAR2DD(E488Q/T375G)-delta-984-1090 (Addgene plasmid #103 871).

### Transfections experiments

E198K NSCs were plated at a density of 100 000 cells/cm^2^ and grown on poly-L-ornithine (PLO) and laminin-coated plates in Neurobasal Media (Life Tech #21103049) supplemented with GlutaMAX Supplement (100X) (Life Tech #35050061), MEM non-essential amino acids solution (100X) (Life Tech #11140050), Pen/Strep, Gibco N-2 Supplement (100X) (Life Tech # 17502048), 20 ng/mL recombinant human FGF basic/FGF2/bFGF (R&D Systems #233-FB-025), 20 ng/mL human EGF recombinant (R&D Systems #236EG200), serum-free B-27 supplement (50X) (Life Tech #17504044), 10 mg/mL insulin solution human recombinant (Sigma Aldrich #SIAL-I9278-5ML) and D-glucose (Life Tech #A2494001). NSC lines were supplemented with Y-27632 RHO/ROCK pathway inhibitor (STEMCELL Technologies #72304) for 24 hours before a complete media change. 24 hours post-seeding, NSCs were transfected with 1 µg plasmid DNA and lipofectamine stem (Life Technologies) following the manufacturer’s guidelines. HEK 293T cells were grown to 80% confluency in DMEM high glucose, 10% fetal bovine serum (FBS), and 1% L-glutamine. Medium was replaced 24 hours post-transfections and 48 hours post-transfection cells were washed with PBS and lysed using TriZol Reagent. Experiments were performed in biological triplicate.

### Amplicon sequencing

To determine the relative read number of healthy and mutant transcripts or percent adenosine editing, RNA extractions were performed on not-treated and treated samples with the Direct-zol RNA Miniprep kit (Zymo Research) and subsequently used for reverse transcription with RevertAid First Strand cDNA Synthesis Kit. Polymerase chain reaction (PCR) amplification was performed on cDNA with amplicon primers containing a 5ʹ barcode sequence of 5 base pairs. Following the PCR reaction, a PCR cleanup was performed and equal concentrations of samples were pooled for Illumina sequencing by the CCIB MGH DNA Core. FASTQ files were analyzed for the number of reads reflecting the wild-type exon 5 (GAA) and mutant exon 5 (AAA) sequences. Percent editing of treated groups was calculated as the number of mutant transcripts over wild-type transcripts and normalized to not-treated.

## Statistical analysis

Statistical analyses were performed in Prism 8 (GraphPad Software) and represented as the mean ± SD. Molecular assessments were performed in biological triplicates for qPCR experiments. Cellular assessments on morphology were performed across 3 independent passages with biological groups of 3-6 (NeuroCult) or groups of 12 (STEMdiff Midbrain) and microscopy images are depicted as a representative of the cell culture. Genome-wide assessments were performed in at least duplicates as noted. Between-group differences were analyzed using a One-way analysis of variance (ANOVA). Statistical differences between the means of the 2 groups were determined using an independent samples *t*-test. The *P* value cutoff for all targeted analyses was set at .05 for all analyses. The null hypothesis was rejected for tests with FDR < 5%. Statistical analyses of differentially expressed genes were performed using DESeq2 in R Studio 3.6.0. The null hypothesis was rejected for tests with FDR < 1%.

## Results

### Generation of patient-derived E198K iPSC and isogenic control iPSC lines

**Figure 1.**
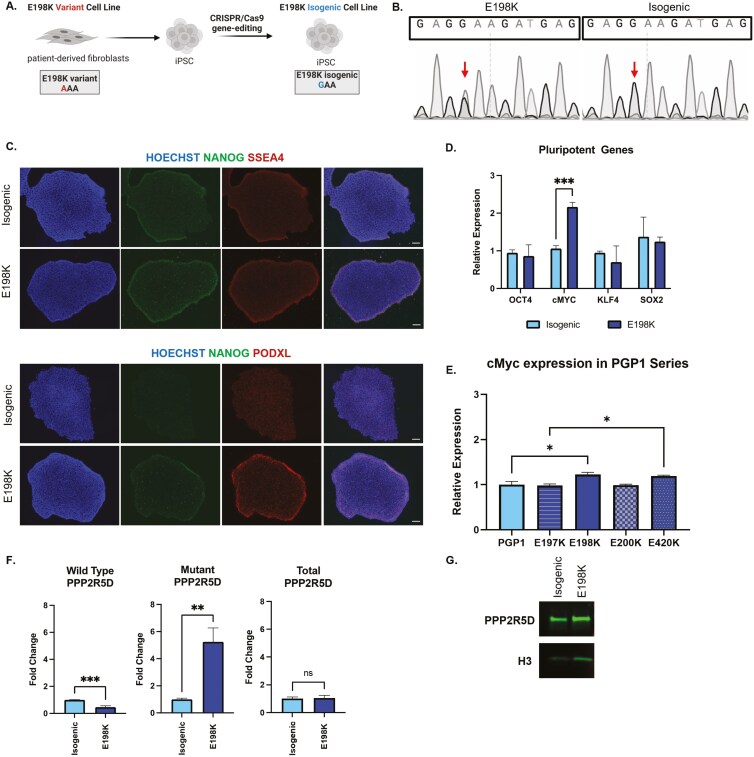
Generation of patient-derived E198K and isogenic iPSC lines. (A) Graphic of E198K fibroblast line reprogrammed to iPSCs to create E198K iPSC line and CRISPR/Cas9 editing of E198K variant to create isogenic line. (B) Sanger sequencing of *PPP2R5D* exon 5 confirms heterozygous guanosine and adenosine peaks in E198K line (left) and correction in the isogenic line (right). (C) Representative 10× images of isogenic and E198K iPSCs positive for pluripotent markers. Scale bar represents 100 µM. (D) Relative expression of canonical pluripotent genes in isogenic and E198K iPSCs determined by RT-qPCR. *** Significantly different from isogenic iPSCs, 2-way ANOVA *P* < .05. (E) Relative expression of *cMYC* in *PPP2R5D* allele iPSC series determined by RT-qPCR. * Significantly different from healthy PGP iPSCs, *n* = 3 biological replicates (dot) per line, one-way ANOVA *P* = .0048. (F) Wild-type and mutant PPP2R5D in isogenic and E198K iPSCs determined by RT-qPCR with allele-specific primers. ***Significantly different from isogenic iPSC, *t* test *P* = .0007. **Significantly different from isogenic PPP2R5D, *t* test *P* = .0021. (G) Western blot of PPP2R5D (B56δ) in isogenic and E198K iPSCs with Histone 3 (H3) housekeeping.

Fibroblasts from a patient harboring a de novo heterozygous missense mutation in *PPP2R5D* (E198K) were reprogrammed to iPSCs using Sendai virus and genomic stability was confirmed with karyotyping analysis. The E198K variant was corrected with CRISPR/Cas9 to create an isogenic control iPSC line (Figure 1A). Sanger sequencing confirmed the heterozygous mutation at the E198K site in the patient-derived line and the correction of the heterozygous mutation in the isogenic line (Figure 1B). The isogenic and E198K iPSC lines were positive for pluripotent markers such as *NANOG*, *SSEA4,* and PODXL and developed uniform pluripotent colonies with defined borders (Figure 1C). We did not observe differences in gene expression for pluripotency genes *OCT4*, *KLF4,* and *SOX2* but identified a higher level of *cMYC* expression (*P* = .0005) in the E198K iPSC line relative to the isogenic control (Figure 1D). Elevated *cMYC* expression was also observed in E198K and E420K iPSCs where the PPP2R5D mutation was introduced into a healthy iPSC parental line, suggesting *cMYC* dysregulation may be distinct to 2 clinically severe genotypes of Jordan syndrome (Figure 1E). An allele-specific quantitative polymerase chain reaction (qPCR) for wild-type and mutant *PPP2R5D* confirmed expression of the mutant allele in the E198K iPSC line (*P *= .0021) but not the isogenic iPSC line (*P *= .0007) (Figure 1F). In order to determine if the E198K variant affects *PPP2R5D* at the protein level, we confirmed the presence of B56δ in the E198K and isogenic lines (Figure 1G).

### Neural stem cell marker expression and proliferation is reduced in Jordan syndrome E198K iPSCs induced into a neural fate

**Figure 2.**
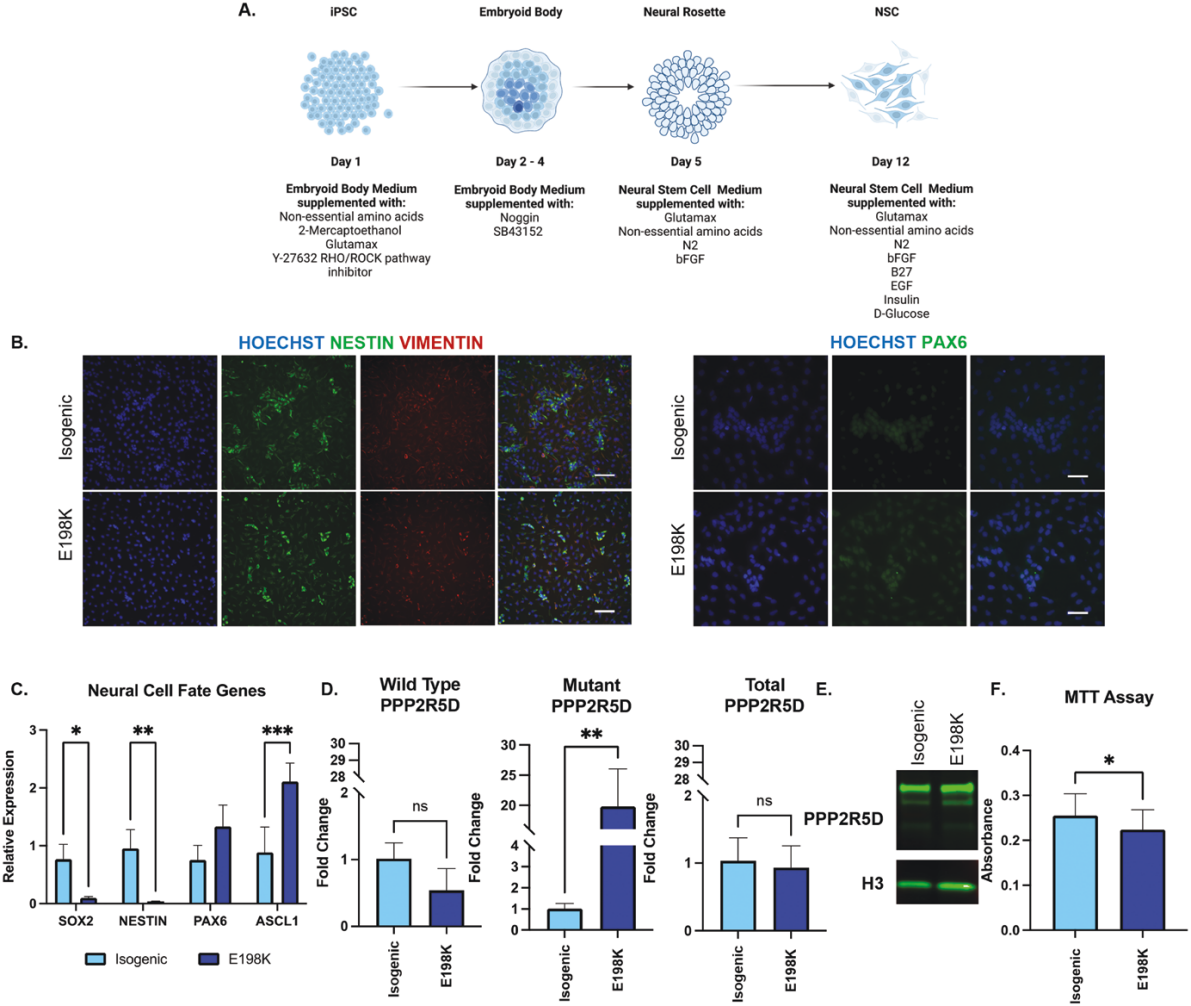
Neural stem cell marker expression and proliferation is reduced in Jordan syndrome E198K iPSCs induced into a neural fate. (A) Graphic of iPSC induction to a neural stem cell fate. (B) Representative 20× images of isogenic and E198K NSCs positive for neural stem cell markers. Scale bar represents 100 µM. (C) Relative expression of neural cell fate genes in isogenic and E198K iPSCs determined by RT-qPCR. *Significantly different from isogenic NSCs, 2-way ANOVA *P* < .05. (D) Wild-type and mutant PPP2R5D in isogenic and E198K NSCs determined by RT-qPCR with allele-specific primers. **Significantly different from isogenic PPP2R5D, *t* test *P *= .0064. (E) Western blot of PPP2R5D (B56δ) in isogenic and E198K NSCs with Histone 3 (H3) housekeeping. (F) Proliferation analysis on isogenic and E198K NSCs (*n* = 22 biological replicates (dots) isogenic and *n* = 20 biological replicates (dots) per line. *Significantly different from isogenic neurons, *n* = 3 independent experiments, *t*-test *P* = .0352.

iPSC lines were differentiated into a neural lineage over a 12-day-period by generating embryoid bodies and subsequently neural rosettes (Figure 2A). Isogenic and E198K NSCs were positive for NESTIN, VIMENTIN, and PAX6, confirming a neural stem cell fate (Figure 2B). We then assessed the expression of early neural fate genes such as *SOX2*, *NESTIN*, *PAX6,* and *ASCL1* with qPCR. Here, we observed higher expression of stem and progenitor markers such as *SOX2* (*P* = .0456) and *NESTIN* (*P* = .0049) in the isogenic line. *ASCL1*, a master regulator for neurogenesis was significantly higher in the E198K NSCs (*P* = .0003) (Figure 2C). Allele-specific qPCRs in the NSCs confirmed no difference in wild-type *PPP2R5D* expression between the isogenic and E198K NSC lines. Mutant *PPP2R5D* expression was higher in the E198K NSC line (*P* = .0064) compared to the isogenic line (Figure 2D). Western blot with NSC lysate confirmed B56δ presence in E198K and isogenic lines (Figure 2E). Interestingly, E198K NSCs displayed a reduction in proliferation compared to isogenic NSCs (*P* = .0352) (Figure 2F). A reduction in proliferative abilities paired with reduced neural stem cell marker expression and higher pro-neuronal expression suggests the E198K mutation may disrupt stem cell differentiation and proliferation, thus contributing to dysregulation in neurogenesis.

### E198K midbrain neurons are more abundant and complex than isogenic midbrain neurons

**Figure 3.**
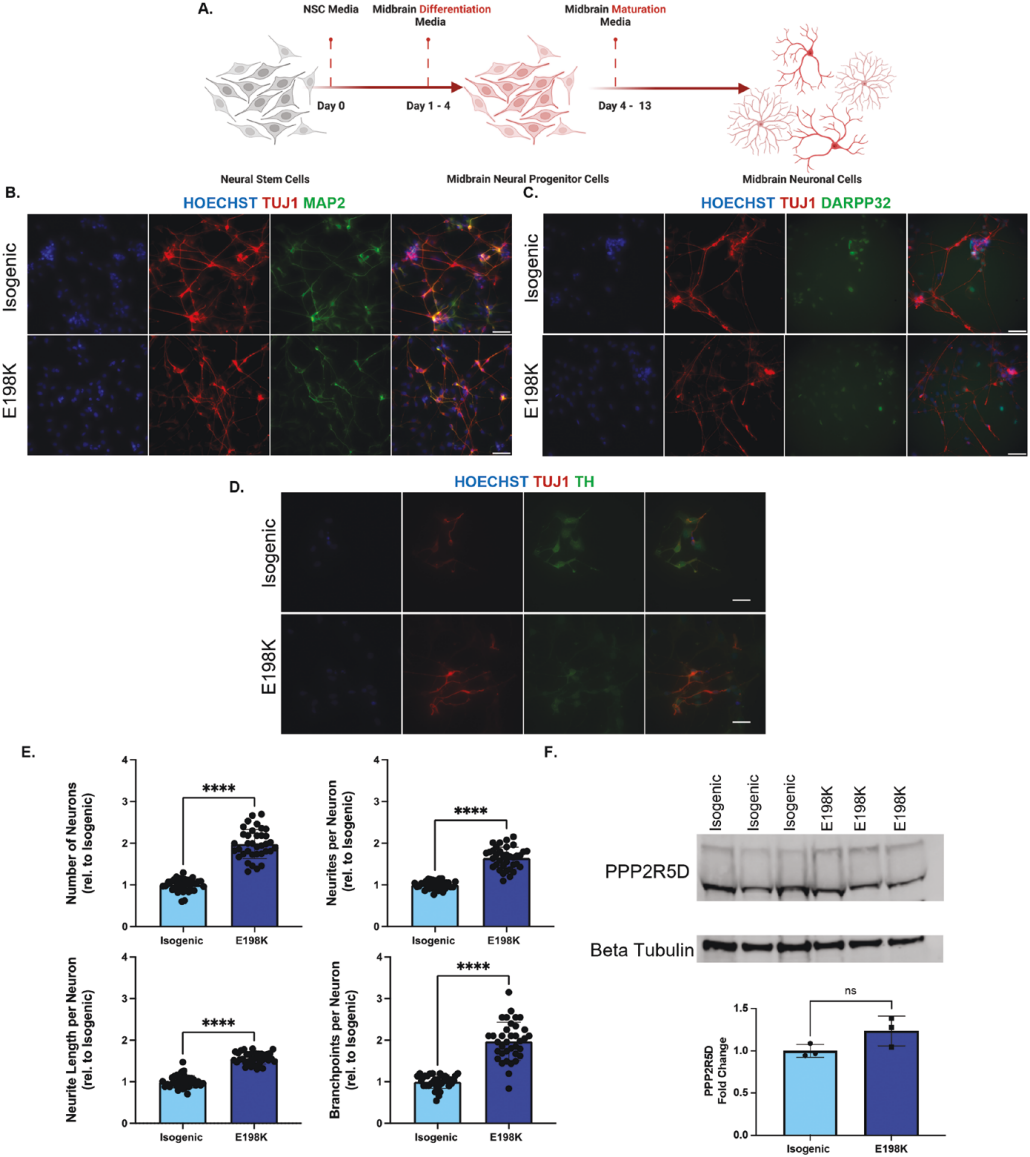
E198K midbrain neurons are more abundant and complex than isogenic midbrain neurons. (A) Graphic of timeline to create midbrain NSCs and midbrain neurons. (B-D) Representative 20× images of isogenic and E198K neurons positive for neuronal markers and midbrain markers. Scale bar represents 100 µM. (E) Neuronal morphology analysis on isogenic and E198K neurons at day 13 (*n* = 36 biological replicates (dots) per line over 3 independent experiments. ****Significantly different from isogenic midbrain neurons, *n* = 3 independent experiments, *t*-test *P* < .05. (F) Western blot of PPP2R5D in midbrain neurons.

To investigate the underlying mechanisms contributing to EOPD phenotypes and to determine if neurogenesis is altered in specific neuronal subtypes, we differentiated NSCs into midbrain NSCs and midbrain neurons (Figure 3A). B56δ was detected in midbrain NSCs as well as tyrosine hydroxylase (TH), the rate-limiting enzyme involved in dopamine synthesis. Neither B56δ nor TH protein levels were different between isogenic and E198K midbrain NSCs (Supplementary Figure S1). Midbrain neurons were positive for TUJ1 and MAP2 and expressed midbrain markers, DARPP32 and TH (Figure 3B-D). Morphological analysis revealed a higher number of E198K neurons based on neuronal marker expression and morphology (*P *< .0001). The E198K midbrain neurons also displayed significantly more neurites and branchpoints as well as an increase neurite length (*P *< .0001) (Figure 3E). B56δ was detected in midbrain neurons; however, no differences in protein levels were observed between E198K and isogenic neurons (Figure 3F). NSCs were also differentiated into neurons of a cortical fate over 23 days to understand if the E198K variant impacts neurogenesis across different neuronal subtypes and to represent a brain region associated with ID (Supplementary Figure S2). Isogenic and E198K neurons formed interconnected networks that were positive for TUJ1 and MAP2. A higher number of E198K neurons (*P *< .0001) but no difference in the number of neurites, neurite length, or number of branchpoints was observed. These findings may suggest a dysregulated neuronal differentiation phenotype that can impact neuronal morphology in specific neuronal subtypes.

### Transcriptome-wide changes observed across disease context and cell state

**Figure 4.**
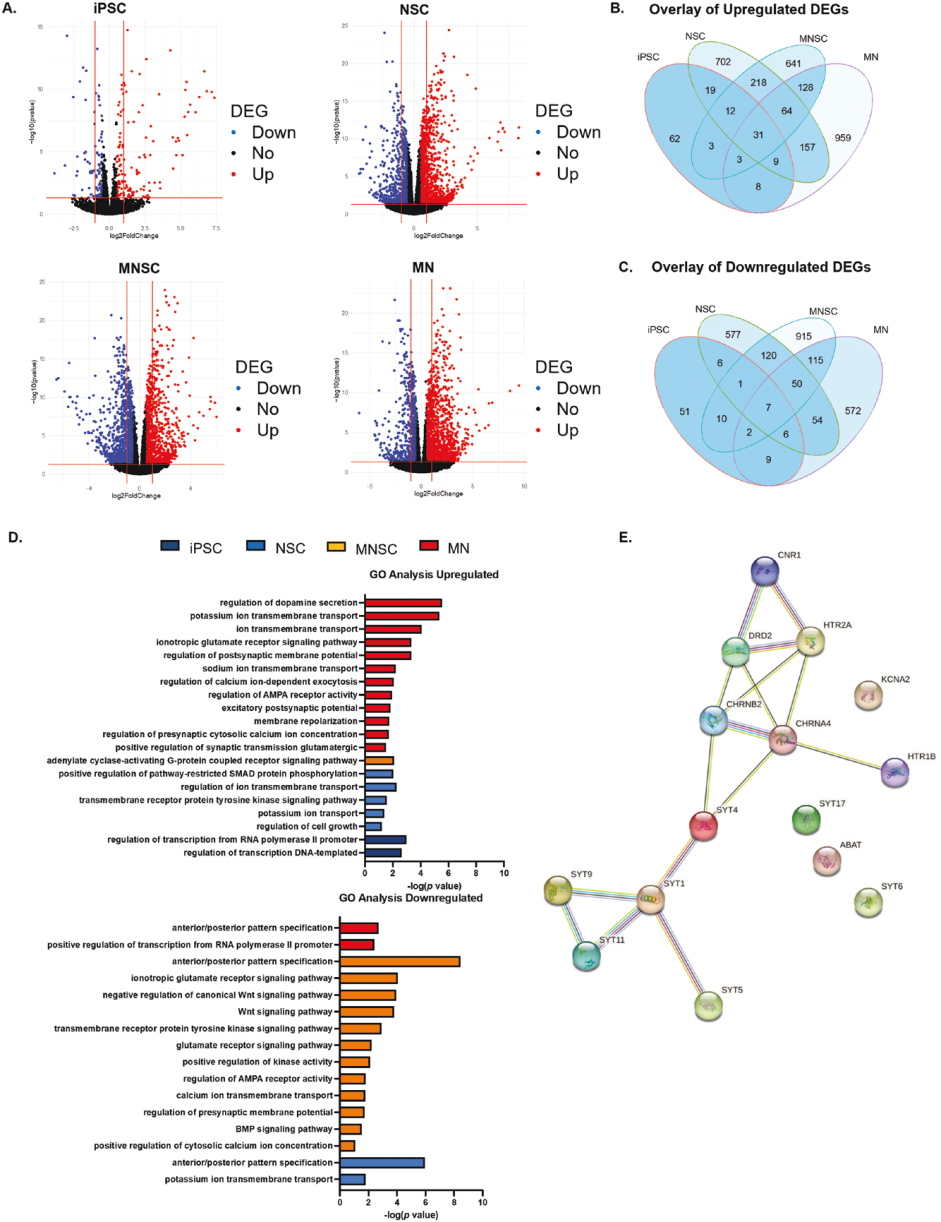
Transcriptome-wide changes across disease context and cell state. (A) Volcano plot of FDR adjusted *P* value vs fold change for differential DESeq2 expression analysis between the isogenic and E198K in iPSC, NSC, midbrain NSCs (MNSC), and midbrain neurons (MN). Differentially expressed genes are highlighted in blue (downregulated) or red (upregulated) based on FDR < 1%, log fold change > 0.5. (B-C) Venn diagram showing the overlap of differentially expressed genes between the iPSC, NSC, MNSC, and MN cell states. (D) Top biological processes upregulated or downregulated in isogenic or E198K iPSCs, NSCs, mNSC, or MN based on FDR adjusted *P* values are shown (E) STRING analysis of genes associated with the regulation of dopamine secretion in MN.

To develop a better understanding of the molecular consequence of mutant *PPP2R5D* pathology, RNA sequencing was performed across the 4 cell states of our disease model (iPSC, NSC, midbrain NSC, and midbrain neuron). A total of 693 differentially expressed genes (DEGs) were observed between isogenic and E198K iPSCs (456 downregulated and 237 upregulated). Pairwise comparisons between the isogenic and E198K cell state revealed 5836 DEGs in the NSCs (3584 downregulated and 2252 upregulated), 4450 DEGs in the midbrain NSCs (1989 downregulated and 2461 upregulated), and 3583 DEGs in midbrain neurons (1706 downregulated and 1877 upregulated) (Figure 4A). A fold-change cutoff of 0.5 was applied for subsequent analysis with the RNA sequencing data. The midbrain NSC cell state had the greatest number of DEGs (1220 downregulated and 1100 upregulated) based on a fold-change cutoff 0.5, suggesting that mutant *PPP2R5D* has a broad effect on the transcriptome during early midbrain development in vitro.

An overlay of genes up or downregulated in the isogenic vs E198K line revealed 31 upregulated and 7 downregulated DEGs shared across the 4 cell states. In addition, there are DEGs between the variant and isogenic line that are unique to a given cell state in this disease model (Figure 4B and C). GO term analysis showed pathways associated with SMAD-protein phosphorylation and ion transport were enriched in NSCs and midbrain NSCs. As E198K cells progress through NSCs, midbrain NSCs, and midbrain neuronal stages, downregulation of genes associated with anterior/posterior pattern specification is observed compared to isogenic cells (Figure 4D). These findings and downregulated Wnt and BMP signaling pathways in E198K midbrain NSCs suggest molecular networks associated with neurogenesis, proliferation, and cell-fate commitment are impacted by mutations in *PPP2R5D*. GO term analysis also revealed an upregulation of genes associated with neuronal pathways important for neuronal firing such as postsynaptic membrane potential, regulation of AMPA receptor activity, potassium and sodium transport, calcium-dependent exocytosis, and membrane repolarization in E198K midbrain neurons. In addition, 14 genes associated with the regulation of dopamine secretion were significantly upregulated in E198K midbrain neurons. Among these genes were the synaptotagmin family (*SYT1*, *SYT17*, *SYT5*, *SYT11*, *SYT4*, *SYT6,* and *SYT9*), receptors involved in neurotransmission (*CHRNA4*, *HTR2A*, *HTR1B*, *DRD2*, *CHRNB2*, *GRM2*) and potassium transport (*KCNA2*). We confirmed the association of these genes in the midbrain neurons by using the STRING database and demonstrated that 11/14 of these genes upregulated in midbrain neurons are known to interact in the regulation of dopamine secretion (Figure 4E). GO term analysis for genes downregulated in the iPSC cell state was not statistically significant as a total of 92 genes were differentially expressed following a fold-change cutoff of 0.5, suggesting an inefficient number of genes to categorize by biological process.

**Figure 5.**
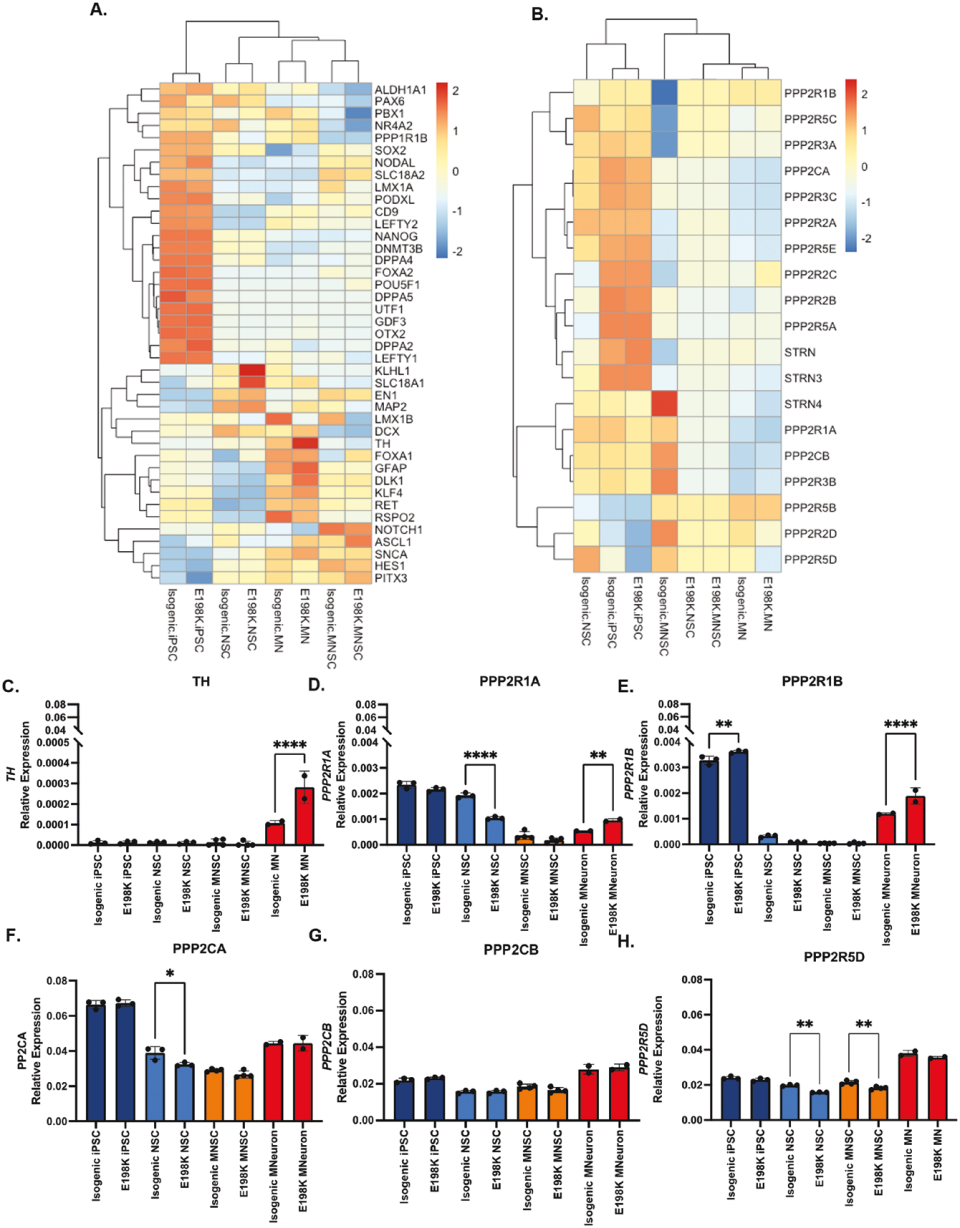
Markers of cell state expression and PP2A Components. (A-B) Heatmap of genes associated with cell state and the PP2A heterotrimeric complex. (C-H) Relative expression based on the number of reads per gene normalized to actin of (C) TH **** significantly different from isogenic midbrain neurons, one-way ANOVA *P* < .0001. (D) PPP2R1A **** Significantly different from isogenic NSCs, one-way ANOVA *P* < .0001 and different from isogenic midbrain neuron *P* = .0035. (E) PPP2R1B **significantly different from isogenic iPSC, one-way ANOVA *P* = .0038 and different from isogenic midbrain neuron *P* < .001. (F) PPP2CA *significantly different from isogenic NSCs, one-way ANOVA *P* < .0110. (G) PPP2CB. (H) PPP2R5D **significantly different from isogenic NSCs, one-way ANOVA *P* < .0001 and different from isogenic midbrain NSC *P* = .0023.

Hierarchical clustering based on the expression of canonical cell state genes showed that both isogenic and E198K cells were able to differentiate into appropriate cell states, suggesting that the E198K variant did not impair differentiation ability (Figure 5A). While no difference in TH protein was detected in isogenic and E198K midbrain NSCs (Supplementary Figure S2), E198K midbrain neurons display higher expression of TH compared to isogenic midbrain neurons (*P *< .0001) (Figure 5C). To understand the impact of the E198K variant on the PP2A holoenzyme components, hierarchical clustering across the various cell states was conducted (Figure 5B). The PP2A holoenzyme contains 3 subunits that display tissue-specific expression patterns. The expression pattern of genes associated with the A subunit (*PPP2R1A* and *PPP2R1B*) was higher in midbrain neurons (*P* = .0035 and *P* < .001) (Figure 5D-E). Of the catalytic C subunit genes (*PPP2CA* and *PPP2CB*), *PPP2CA* expression was reduced in E198K NSCs *(P* = .0110) (Figure 5F-G). While 9 of the B subunits are known to display expression in the brain, we observed a reduction in *PPP2R5D* expression specifically in E198K NSCs (*P* < .0001) and midbrain NSCs (*P* = .0023) (Figure 5H). In the midbrain neurons, the expression of *PPP2R5D* does not change suggesting dysregulation could be due to a change in *PPP2R5D* or PP2A-B56δ function and not changes in *PPP2R5D* expression (Figure 5H).

### Length adjustments of dsRNA substrate and A-C mismatch position improves A > G editing of the E198K site

**Figure 6.**
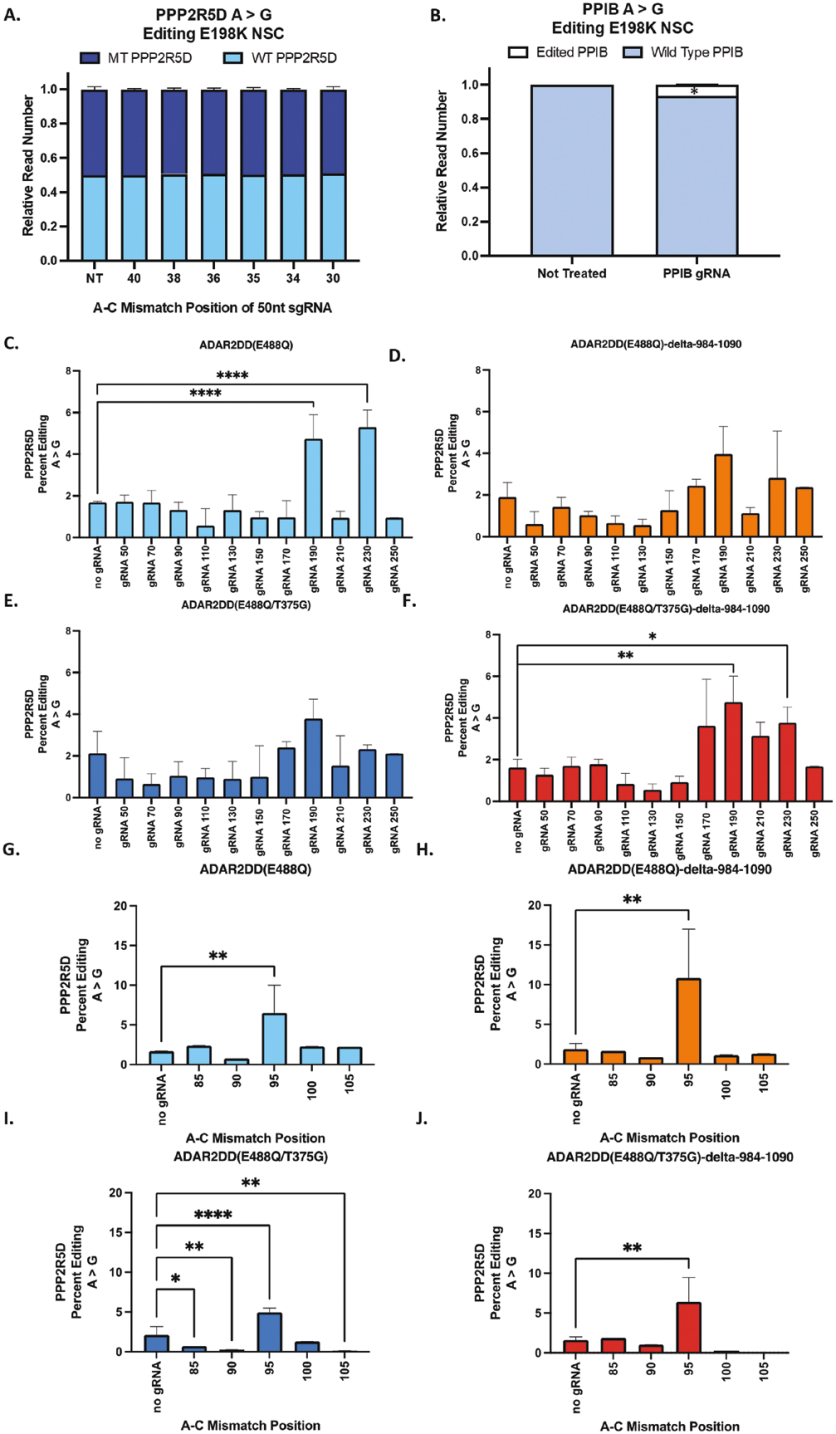
50nt PspdCas13b sgRNA with Tiled A-C Mismatch Do Not Edit the Endogenous E198K site in patient-derived neural stem cells. (A) Amplicon sequencing results of the relative read number of wild-type (GAA) and E198K (AAA) reads in E198K NSCs transfected with sgRNA and dPspCas13b--ADAR2DD(E488Q/T375G)-delta-984-1090 plasmid construct. (B) Amplicon sequencing results of E198K NSCs transfected with PPIB sgRNA and dPspCas13b--ADAR2DD(E488Q/T375G)-delta-984-1090 plasmid construct. Percent editing was determined as the number of edited transcript reads over not edited transcripts in the treated group and normalized to not-treated E198K NSCs. *significantly different from not treated E198K NSCs, t-test *P* < .05. (C-F) Amplicon sequencing results of percent editing of the E198K site in E198K NSCs following screen with sgRNA of increasing length and PspdCas13b-ADAR2DD constructs. Percent editing was determined as the number of edited transcript reads over not edited transcripts in treated group and normalized to not-treated E198K. * ** and **** significantly different from respective construct with no gRNA control, one-way ANOVA *P* < .05. (G—J) Amplicon sequencing results of percent editing of the E198K site in E198K NSCS following screen with sgRNA 190 and variable A-C mismatch position and PspdCas13b-ADAR2DD constructs. Percent editing was determined as the number of edited transcript reads over not edited transcripts in treated group and normalized to not-treated E198K. * ** and **** significantly different from respective construct with no gRNA control, one-way ANOVA *P* < .05.

To validate the dCas13b-ADAR2DD as a site-directed RNA editor (SDRE) of endogenous nucleotides in a patient-derived model, we designed *Psp*dCas13b sgRNA of 50nt in length to correct the E198K pathogenic adenosine nucleotide with a cytosine nucleotide opposing the target adenosine nucleotide (A-C mismatch).^27^ E198K sgRNA was screened via transfection in the patient-derived E198K NSC line. Amplicon sequencing revealed no change in the relative number of mutant or wild-type *PPP2R5D* transcripts compared to not-treated NSCs, suggesting a 50nt sgRNA with variable A-C mismatch positions is not sufficient to edit the E198K variant (Figure 6A). Since ADAR2 requires a double-stranded RNA substrate (dsRNA) for A-to-I conversion, a 50nt sgRNA could be an inefficient length to create a dsRNA substrate for ADAR2 editing. We observed 6.48% (*P *= .0009) editing of the *PPIB* site in the E198K NSCs with a published control sgRNA (Figure 6B). To better understand the transcript-specific constraints and to increase editing efficiency with a CRISPR RNA editor, we screened 4 published *Psp*dCas13-ADAR2DD plasmid constructs at the *PPIB* site in HEK 293T cells (Supplementary Figure S3).^27^ The originally developed *Psp*dCas13b-ADAR2DD(E488Q) construct achieved 15.79% editing (*P* < .0001), while the *Psp*dCas13b truncated version (*Psp*dCas13b-ADAR2DD(E488Q)-delta-984-1090) achieved 8.28% editing (*P* < .0001) and the redesigned higher specificity *Psp*dCas13b-ADAR2DD(E488Q/T375G) achieved 1.24% editing (*P* < .0046) when compared to not-treated E198K NSCs. Interestingly, the AAV-optimized *Psp*dCas13b-ADAR2DD(E488Q/T375G)-delta-984-1090 did not significantly edit the endogenous PPIB site in HEK293T cells (Supplementary Figure S3). The same screen in the E198K NSCs revealed a 0.73% (*P* < .0001) and 1.37% editing (*P* < .0001) at the PPIB site by the *Psp*dCas13b-ADAR2DD(E488Q) and *Psp*dCas13b-ADAR2DD(E488Q)-delta-984-1090, respectively (Supplementary Figure S3). Together, these findings show that endogenous editing is influenced by cell type and percent editing is variable across published *Psp*dCas13b-ADAR2DD constructs.

Next, we performed a *Psp*dCas13b-ADAR2DD construct screen with longer sgRNA targeting the *PPP2R5D* transcript. Rather than design sgRNA with a fixed length and variable A-C mismatches, we sought to determine the optimal dsRNA substrate length for *Psp*dCas13b-ADAR2DD editing of the target transcript. Here, the A-C mismatch was positioned directly in the center of the sgRNA with the sgRNA varying from 50nt to 250nt in length. To better understand the editing efficiency of longer sgRNA with each *Psp*dCas13b-ADAR2DD construct we assessed the percent editing compared to not-treated E198K NSCs. sgRNA of 190nt in length paired with the *Psp*dCas13b-ADAR2DD(E488Q) or *Psp*dCas13b-ADAR2DD(E488Q/T375G)-delta-984-1090 edited the E198K site at 4.73% (*P *< .0001) or 4.75% (*P *< .0011), respectively (Figure 6C and D). sgRNA of 230nt in length and *Psp*dCas13b-ADAR2DD(E488Q) edited at 5.30% (*P *< .0001), while the same sgRNA with *Psp*dCas13b-ADAR2DD(E488Q/T375G)-delta-984-1090 edited at 3.77% (*P *< .0332) (Figure 6C and 6F). E198K NSCs transfected with any sgRNA length *Psp*dCas13b-ADAR2DD(E488Q)-delta-984-1090 or dPspCa13b-ADAR2DD(E488Q/T375G) did not achieve significant editing of the pathogenic adenosine nucleotide (Figure 6E and F). These findings suggest longer sgRNA creates an optimal dsRNA substrate for ADAR2DD to facilitate adenosine-to-inosine editing of the E198K site.

**Figure 7.**
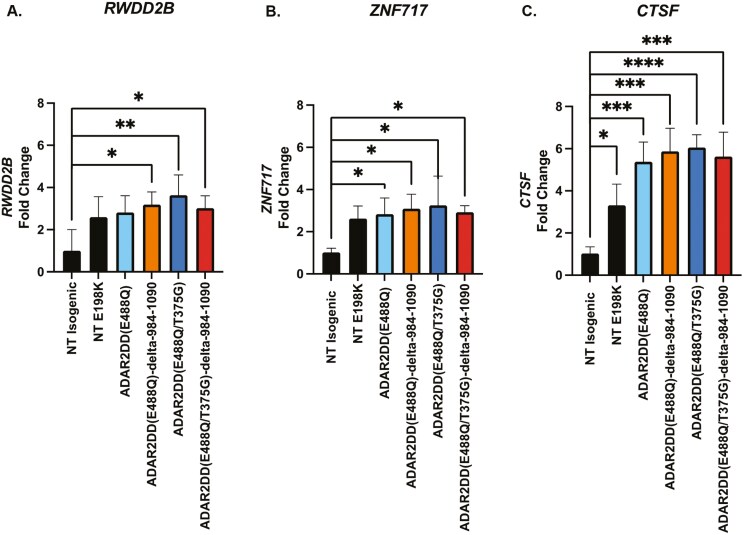
Top DEGs are not rescued by PspdCas13b-ADAR2DD constructs and sgRNA 190. Relative expression of the 3 top DEGs between the isogenic and E198K NSC cell states in isogenic, not-treated E198K NSCs, E198K NSCs treated with *Psp*dCas13b-ADAR2DD(E488Q), *Psp*dCas13b-ADAR2DD(E488Q)-delta-984-1090, *Psp*dCas13b-ADAR2DD(E488Q/T375G), or *Psp*dCas13b-ADAR2DD(E488Q/T375G)-delta-984-1090. (A) *RWDD28* relative expression determined by RT-qPCR. * Significantly different from total isogenic, one-way ANOVA *P* < .05. (B) ZNF717 relative expression determined by RT-qPCR. * Significantly different from total isogenic, one-way ANOVA *P* < .05. (C) CTSF relative expression determined by RT-qPCR. * Significantly different from total isogenic, one-way ANOVA *P* < .05.

To understand how editing efficiency is influenced by the A-C mismatch position we redesigned sgRNA of 190nt in length by placing the A-C mismatch 5nt or 10nt in the 3ʹ or 5ʹ direction of the original position. The original sgRNA of 190nt with an A-C mismatch at position 95 across all constructs achieved the greatest percent editing of mutant transcripts at a frequency of at least 4.9% A-to-I editing (Figure 6G-J). These findings confirm that an A-C mismatch in the center of a sgRNA of 190nt in length as the required design in targeting the endogenous E198K variant in patient-derived NSCs with CRISPR RNA editors. To evaluate the CRISPR RNA editing approach for the molecular rescue of the top 3 DEGs (*RWDD28*, *ZNF717,* and *CTSF*) between the isogenic and E198K NSCs, we analyzed the E198K NSCs transfected with the *Psp*dCas13b-ADAR2DD constructs and sgRNA 190 (Figure 7A-C). These constructs were unable to reduce *RWDD28*, *ZNF717,* or *CTSF* expression comparable to the isogenic NSCs, suggesting that 10.8% editing of the E198K site does not presently lead to transcriptomic rescue.

## Discussion

The occurrence of NDD or EOPD in individuals with *PPP2R5D* variants suggests a critical role in brain development and health. Indeed, *PPP2R5D* is a brain-enriched regulatory subunit of PP2A and many of the PP2A-B56δ substrates are important in neurons.^13-15^ Previously, there were no human neuronal disease models to understand *PPP2R5D* as an NDD risk gene. Here, we develop and characterize a patient-derived neuronal model to advance knowledge of the underlying molecular and cellular mechanisms. We used a cellular reprogramming approach to create iPSCs from patient-derived fibroblasts harboring the most common *PPP2R5D* variant. We used these iPSC to further develop our in vitro model by subsequently completing differentiations to NSCs and then neurons. Our approach included iPSCs, NSCs and neurons which provided the opportunity to identify disease phenotypes in multiple cell states reflected during neurodevelopment.

The screening of pluripotent markers revealed higher cMYC expression in the E198K iPSCs which was further supported in additional *PPP2R5D* iPSC lines. cMYC regulation is important for normal cell growth and proliferation. Thus, the observation of reduced proliferation in E198K NSCs is interesting. Previous groups have described the importance of PP2A-B56δ regulation of cMYC via GSK3β.^28–30^ cMYC binds and drives transcription of *PPP2R5D* which enables PP2A-B56δ to dephosphorylate GSK3β ser-9 allowing for negative regulation of cMYC by ubiquitination and degradation.^28^ Dysregulation in the metabolic AKT-mTOR signaling pathways has been shown to impact cell growth in a HEK 293T *PPP2R5D* model.^17^ To date, there are no known PP2A-B56δ substrates involved in neurogenesis; however, altered neurogenesis and neuronal maturation are linked to many NDD.^30^ The BMP signaling pathway facilitates stem cell differentiation and proliferation. From a transcriptomic standpoint, the downregulation of genes associated with BMP signaling pathways in our disease model could provide a potential mechanism contributing to reduced proliferation despite increased neurons in a disease state. Future work will need to account for how changes in gene expression influence pathways at large in the E198K disease model by incorporating global proteomic and phosphoproteomic analysis. These studies will also be useful to capture phosphatase and kinase dysregulation across cell states in neurodevelopment.

The occurrence of EOPD phenotypes in individuals with *PPP2R5D* variants is interesting as it is unclear if dysregulation in the dopamine circuit arises in neurodevelopment or early adulthood. We created an in vitro neurodevelopmental model with midbrain NSCs and differentiated to midbrain neurons to better understand *PPP2R5D*-related dopamine dysregulation at a molecular and cellular level. Our work shows successful differentiation to a population of midbrain neurons that express key markers of the midbrain cell-fate and display differences in neuronal complexity between isogenic controls and the E198K cell states. Other groups have reported increased neurites and dendritic branching in alpha-synuclein models of Parkinson’s Disease (PD).^31,32^ A hallmark of PD pathology is a reduction in dopamine release into the striatum following a loss of neurons in the substantia nigra pars compacta.^33^ Our findings suggest EOPD phenotypes associated with *PPP2R5D* dysregulation do not result from neuronal loss since a higher number of E198K midbrain neurons was observed following a 9-day maturation. Future work will need to model disease progression in vitro by extending the maturation time in vitro. Vesicular-mediated transport along the axon to the synapse is also implicated in PD models^69^. Here, we observe differentially expressed genes in the midbrain neurons which are involved in ion transport and calcium-dependent vesicular release. While we have yet to determine if these midbrain neurons are functionally active, secrete, or uptake dopamine, no change in TH protein suggests that the dopamine synthesis pathway is not impacted by the E198K variant. Thus, our in vitro model can be used as a tool to decipher *PPP2R5D*-related molecular and morphological dysregulation during midbrain development.

The consequence of *PPP2R5D* variants across neurodevelopment was previously unknown which led us to apply a corrective approach rather than reducing mutant *PPP2R5D* or upregulating healthy *PPP2R5D*. Here, we evaluate a novel strategy for editing endogenous pathogenic nucleotides at the transcript level. In this system, an sgRNA complementary to the mutant E198K transcript sequence forms a dsRNA substrate to enable *Psp*dCas13b-ADAR2DD binding and deaminase activity. While the 4 *Psp*dCas13-ADAR2DD plasmid constructs were developed to allow for on-target editing with minimal off-target edits and future AAV platforms, these findings support optimization of sgRNA design and RNA editing constructs to edit endogenous sites. We show the importance of *Psp*dCas13b sgRNA design through a combination of length and A-C mismatch position adjustments in targeting the mutant *PPP2R5D* transcript. The initial sgRNA of 50nt in length with variable A-C mismatch positions did not edit the endogenous E198K site in E198K NSCs, despite this sgRNA length and an A-C mismatch in positions 32-36 promoting endogenous *PPIB* editing in HEK293T cells and E198K NSCs. However, we also found 50nt sgRNA with the A-C mismatch in the 25nt position unable to edit the E198K site. These findings confirm the presence of transcript-specific constraints that dictate sgRNA and *Psp*dCas13b-ADAR2DD editing efficiency. While we observe as high as 10.8% editing with sgRNA 190 or 5.3% with sgRNA 230, it is likely that a transient transfection approach hinders us from capturing maximum editing of the E198K site. NDDs arising from heterozygous dominant negative mutations as described here, will likely require a high amount of correction to rescue molecular and cellular dysregulation. Future work will need to account for sustained editing with stable cell lines, selection tags, or a lentivirus-based platform to maximize editing efficiency and enable the selection of cells treated with CRISPR RNA editors.

The adjustments to gRNA length were supported by previous findings that ADAR-recruiting RNA (arRNAs) of 111nt recruited endogenous ADAR2 to edit pathogenic *COL3A1, BMPR2*, *HI1*, *FANCC*, *MYBPC3,* and *IL2RG* in HEK 293T cells, *PPIB* in primary T cells and *IDUA* in patient-derived fibroblasts.^34^ Here, we demonstrate that editing the E198K site with the CRISPR RNA editing approach requires PspdCas13b sgRNA of 190nt, suggesting each transcript and editing approach may have varying sgRNA design requirements. Importantly, we find that placing the A-C mismatch in the center of sgRNA of 190nt resulted in the highest A-to-I conversion and increase in healthy transcripts. In this study, we also evaluated four *Psp*dCas13-ADAR2DD plasmid constructs. The AAV-optimized *Psp*dCas13b-ADAR2DD(E488Q/T375G)-delta-984-1090 construct contains truncated *Psp*dCas13b and an ADAR2DD mutant to enable higher on-target specificity with a reduction in overall on-target and off-target editing. Interestingly, we find that sgRNA of 190nt with an A-C mismatch in position 95 has comparable editing between the original and AAV-optimized ADAR2DD constructs which will be advantageous in the future when translating CRISPR RNA editors in disease-relevant mouse models.

While the longer sgRNA permitted editing, it is important to note that this creates a longer dsRNA substrate which may increase the likelihood of off-target editing due to the frequency of adenosine nucleotides within the sgRNA binding window or in transcripts with partial complementary to the sgRNA.^34–36^ In addition, the propensity for mutations in *PPP2R5D* would suggest that off-target edits would be a significant therapeutic hurdle. The balance between identifying a system that promotes high on-target editing and moderate-low off-target editing is crucial to prevent additional mutations that alter protein function and signal cascades. An advantage to the CRISPR RNA approach is the flexibility with which sgRNA can be modified to minimize off-target editing. Including an A-G mismatch opposing highly edited off-target sites within the sgRNA binding window has been successfully employed with ADAR recruiting RNAs (arRNAs) in RTT primary hippocampal neurons to edit the R106Q variant.^20^ However, how one or multiple A-G mismatches impact sgRNA binding for endogenous editing with *Psp*dCas13b-ADAR2DD has not been explored.

Here, we developed a stem-cell-based disease model showing a molecular and morphological basis for *PPP2R5D*-related EOPD in cells of a midbrain fate. This work identified pathways that are critical for healthy brain development and can be used as biomarkers in subsequent studies. The present work also demonstrated that the novel CRISPR/*Psp*dCas13b fused to the ADAR2DD can be employed for RNA editing in a patient-derived stem cell model and thus allowed for sgRNA screening in a line that harbors the endogenous pathogenic nucleotide. Through sgRNA and *Psp*dCas13b-ADAR2DD screening, we identified *PPP2R5D* transcript parameters that were important for RNA editing at the E198K site.

## Supplementary Material

sxae068_suppl_Supplementary_Material

## Data Availability

To review GEO accession GSE238213: Go to https://www.ncbi.nlm.nih.gov/geo/query/acc.cgi?acc=GSE238213. Enter token qnozakmifhevdsj into the box.
