# Supplementary material for: A de novo missense mutation in PPP2R5D alters dopamine pathways and morphology of iPSC-derived midbrain neurons
Source: Stem Cells. 2024 Oct 26;43(1):sxae068. doi: 10.1093/stmcls/sxae068 (PMC11811633; doi:10.1093/stmcls/sxae068)
Supplement: sxae068_suppl_Supplementary_Material [file sxae068_suppl_supplementary_material.zip › Supplemental fig and Tables/Supplemental Figures and Tables.docx]

**Supplemental Figures:**

**Figure S1.** Neural Stem Cells Induced into a Midbrain Cell State

(A) Western Blot of PPP2R5D in midbrain NSCs. (B) Western Blot of TH in midbrain NSCs. (C) Representative 20X images of isogenic and E198K neurons positive for midbrain marker TH and LMX1B. Scale bar represents 100uM.

**Figure S2.** Isogenic and E198K NSCs Differentiated Into Neurons Do Not Display Differences in Neuronal Complexity.

(A) Graphic of timeline to create neurons from NSCs. (B) Representative 10X images of isogenic and E198K neurons positive for neuronal markers. Scale bar represents 100uM.(C) Neuronal morphology analysis on isogenic and E198K neurons at day 23 (n = 24 biological replicates (dots) isogenic and n = 27 biological replicates (dots) per line over three independent experiments. **** significantly different from isogenic neurons, n = 3 independent experiments, t-test *p* < 0.05

**Figure S3**. Endogenous Editing Efficiency is Influenced by PspdCas13b-ADAR2DD Constructs.

(A) Graphic of PspdCas13b-ADAR2DD constructs used in RNA editing screen. (B) Amplicon sequencing results of percent editing of the PPIB site in HEK 293T following screen with published sgRNA and PspdCas13b-ADAR2DD constructs. Percent editing determined as number of edited transcript reads over not edited transcripts in treated group and normalized to not-treated HEK293T. ** and ****significantly different from not-treated, one-way ANOVA p < 0.05. (C) Amplicon sequencing results of percent editing of the PPIB site in E198K NSCs following screen with published sgRNA and PspdCas13b-ADAR2DD constructs. Percent editing determined as described above. **** significantly different from not-treated, one-way ANOVA p < 0.05.

**Figure S4. PGP1 Series iPSC Lines Display Stemness**

Representative 10X images of PGP1-Healthy, PGP1-E197K, PGP1-E198K, PGP1-E200K, and PGP1-E420K iPSCs positive for pluripotent markers Nanog and SSEA4. Scale bar represents 100uM.

**Supplemental Tables:**

**Table S1:** Primary Antibodies used in Immunocytochemistry Staining

| **Primary Antibody** | **Host** | **Manufacturer** | **Catalog Number** | **Dilution** |
| --- | --- | --- | --- | --- |
| Nanog | Goat | RnD Systems | SC025 | 1/100 for isogenic and E198K ICC  1/200 for PGP1 series ICC |
| SSEA4 | Mouse | RnD Systems | SC025 | 1/100 for isogenic and E198K ICC  1/200 for PG |
| PODXL | Mouse | RnD Systems | SC025 | 1/100 |
| NESTIN | Rabbit | Abcam | 176571 | 1/100 |
| VIMENTIN | Rat | RnD Systems | SC025 | 1/50 |
| TUJ1 | Mouse | Abcam | 78078 | 1/500 |
| MAP2 | Rabbit | Abcam | 254264 | 1/1000 |
| TH | Chicken | Abcam | 76442 | 1/500 |
| DARPP32 | Rabbit | Abcam | 40801 | 1/100 |

**Table S2:** Secondary Antibodies used in Immunocytochemistry Staining

| **Secondary Antibody** | **Host** | **Manufacturer** | **Catalog Number** | **Dilution** |
| --- | --- | --- | --- | --- |
| Alexa Fluor 594, anti-Rabbit IgG (H+L) | Goat | Thermofisher | A11012 | 1/250 |
| Alexa Fluor 594, anti-Mouse IgG (H+L) | Goat | Thermofisher | A11032 | 1/250 |
| Alexa Fluor 488, anti-Rabbit IgG (H+L) | Goat | Thermofisher | A11008 | 1/250 |
| Alexa Fluor 647, anti-Guinea Pig IgG (H+L) | Goat | Thermofisher | A21450 | 1/250 |
| Alexa Fluor 647, anti-Rat IgG (H+L) | Goat | Thermofisher | A21472 | 1/250 |
| Alexa Fluor 647, anti-Rabbit IgG (H+L) | Goat | Thermofisher | A21244 | 1/250 |
| Alexa Fluor 488, anti-Chicken IgG (H+L) | Goat | Thermofisher | A11039 | 1/250 |
| Alexa Fluor 488, anti-Goat IgG (H+L) | Rabbit | Thermofisher | A11078 | 1/250 |

**Table S3:** qPCR Primers

| Gene | Forward Sequence | Reverse Sequence |
| --- | --- | --- |
| GAPDH | AATCCCATCACCATCTTCCA | CTCCATGGTGGTGAAGACG |
| OCT4 | CCCACACTGCAGCAGATCA | CACACTCGGACCACATCCTT |
| CMYC | GTAGTGGAAAACCAGCAGCCT | AAGCTAACGTTGAGGGGCAT |
| KLF4 | CGAACCCACACAGGTGAGAA | GCGAATTTCCATCCACAGCC |
| SOX2 | GCCGAGTGGAAACTTTTGTCG | GCAGCGTGTACTTATCCTTCTT |
| PPP2R5D | TACGAGACGGAGCATCACAACG | GGAAGTAGGACACGGATGAGGA |
| WT PPP2R5D | ACCTTCATCGAATCCCACAG | TCCAGGGTGGGCTCATCTTC |
| MT PPP2R5D | ACCTTCATCGAATCCCACAG | TCCAGGGTGGGCTCATCTTT |
| NESTIN | TCAAGATGTCCCTCAGCCTGGA | AAGCTGAGGGAAGTCTTGGAGC |
| PAX6 | CTGAGGAATCAGAGAAGACAGGC | ATGGAGCCAGATGTGAAGGAGG |
| ASCL1 | CAAGAGAGCGCAGCCTTA | GCAAAAGTCAGTGCTGAACG |
| CTSF | GATGAAGCAAGCCAAGTCTGTGG | CACATTGCCTGTGACTGAGAAGG |
| RWDD28 | TATGAACCTGGATGTATCTGACGA | TATGAACCTGGATGTATCTGACGA |
| ZNF717 | GAGACCTACAGCAGCCTGGTAT | CTATCCATGGCTCTGCTCCTTG |

**Table S4:** Primary antibodies used in western blots

| **Primary Antibody** | **Host** | **Manufacturer** | **Catalog Number** | **Dilution** |
| --- | --- | --- | --- | --- |
| PPP2R5D | Rabbit | Abcam | 188323 | 1/1000 |
| Beta Actin | Mouse | Abcam | 6276 | 1/1500 |
| Tyrosine Hydroxylase | Rabbit | Abcam | 76442 | 1/1000 |

**Table S5:** Secondary antibody used in western blots

| **Secondary Antibody** | **Host** | **Manufacturer** | **Catalog Number** | **Dilution** |
| --- | --- | --- | --- | --- |
| Anti-rabbit | Donkey | IRDye | 926-32213 | 1/2000 |
| Anti-mouse | Goat | IRDye | 926-68070 | 1/2000 |

**Table S6: sgRNA sequences used in this study**

**Table S7: PspdCas13b-ADAR2DD constructs used in this study**
