# Supplementary figures and images for: A de novo missense mutation in PPP2R5D alters dopamine pathways and morphology of iPSC-derived midbrain neurons
Source: Stem Cells. 2024 Oct 26;43(1):sxae068. doi: 10.1093/stmcls/sxae068 (PMC11811633; doi:10.1093/stmcls/sxae068)

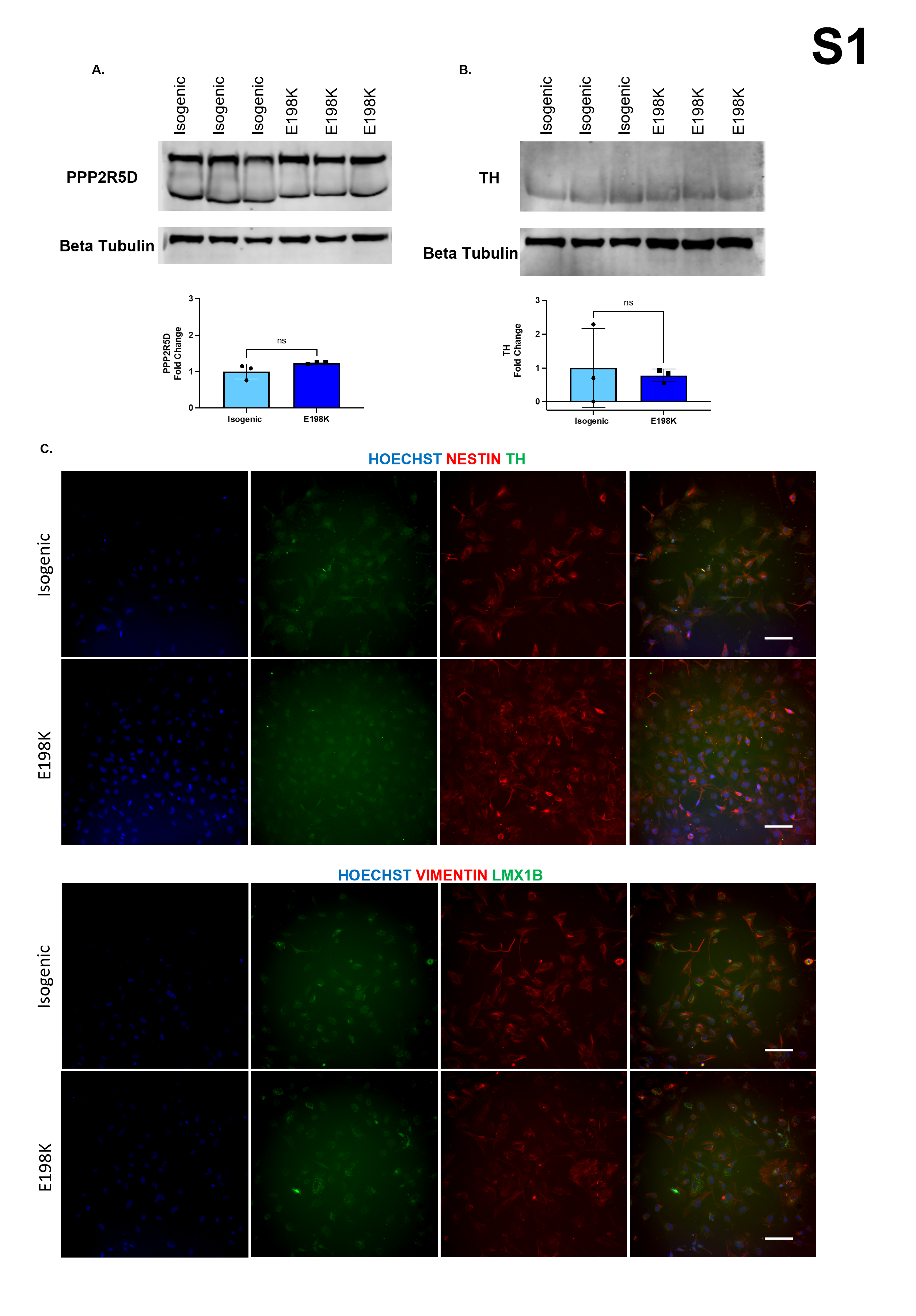

Supplement: sxae068_suppl_Supplementary_Material [file sxae068_suppl_supplementary_material.zip › Supplemental fig and Tables/SFig 1.png]

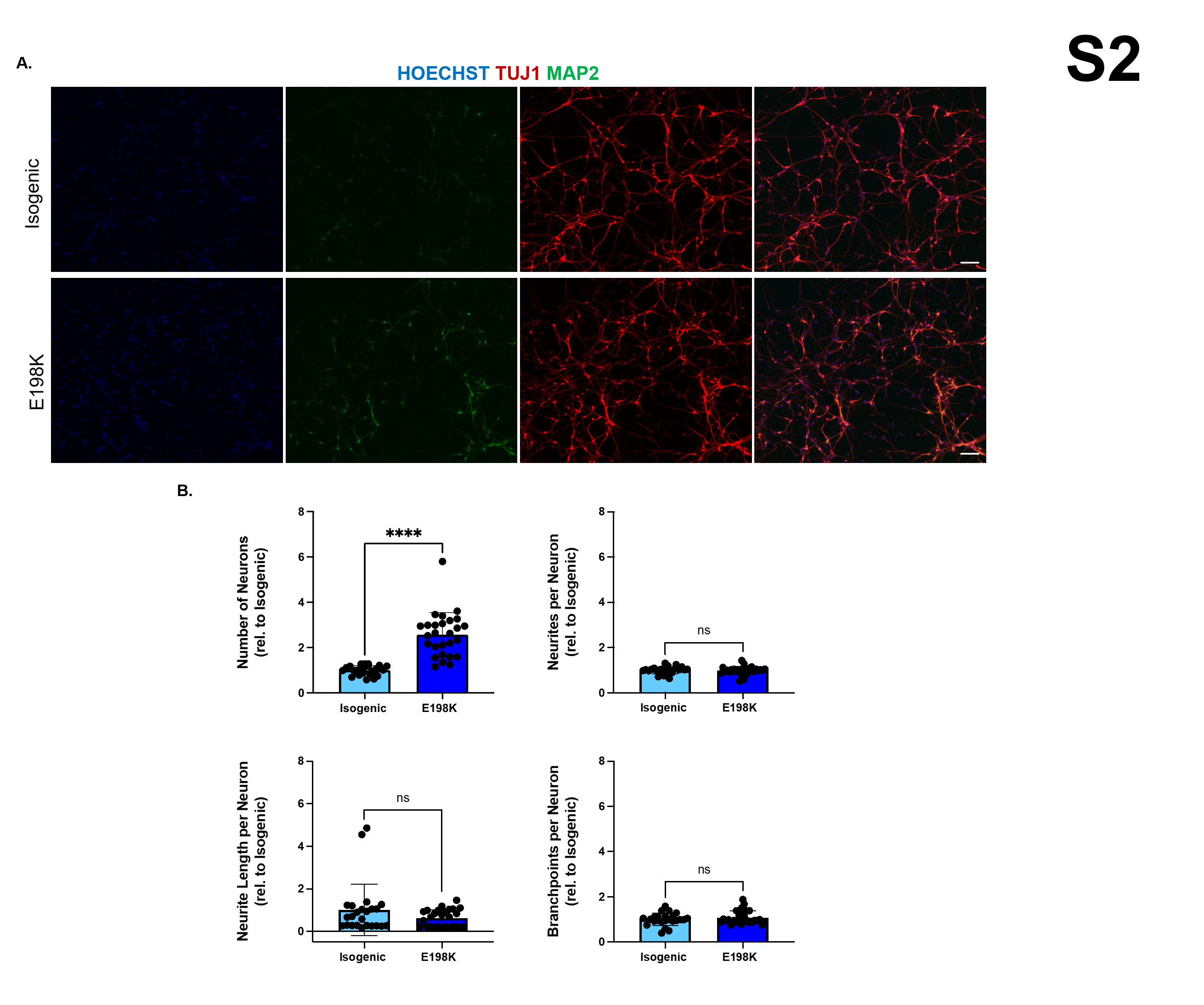

Supplement: sxae068_suppl_Supplementary_Material [file sxae068_suppl_supplementary_material.zip › Supplemental fig and Tables/SFig 2.png]

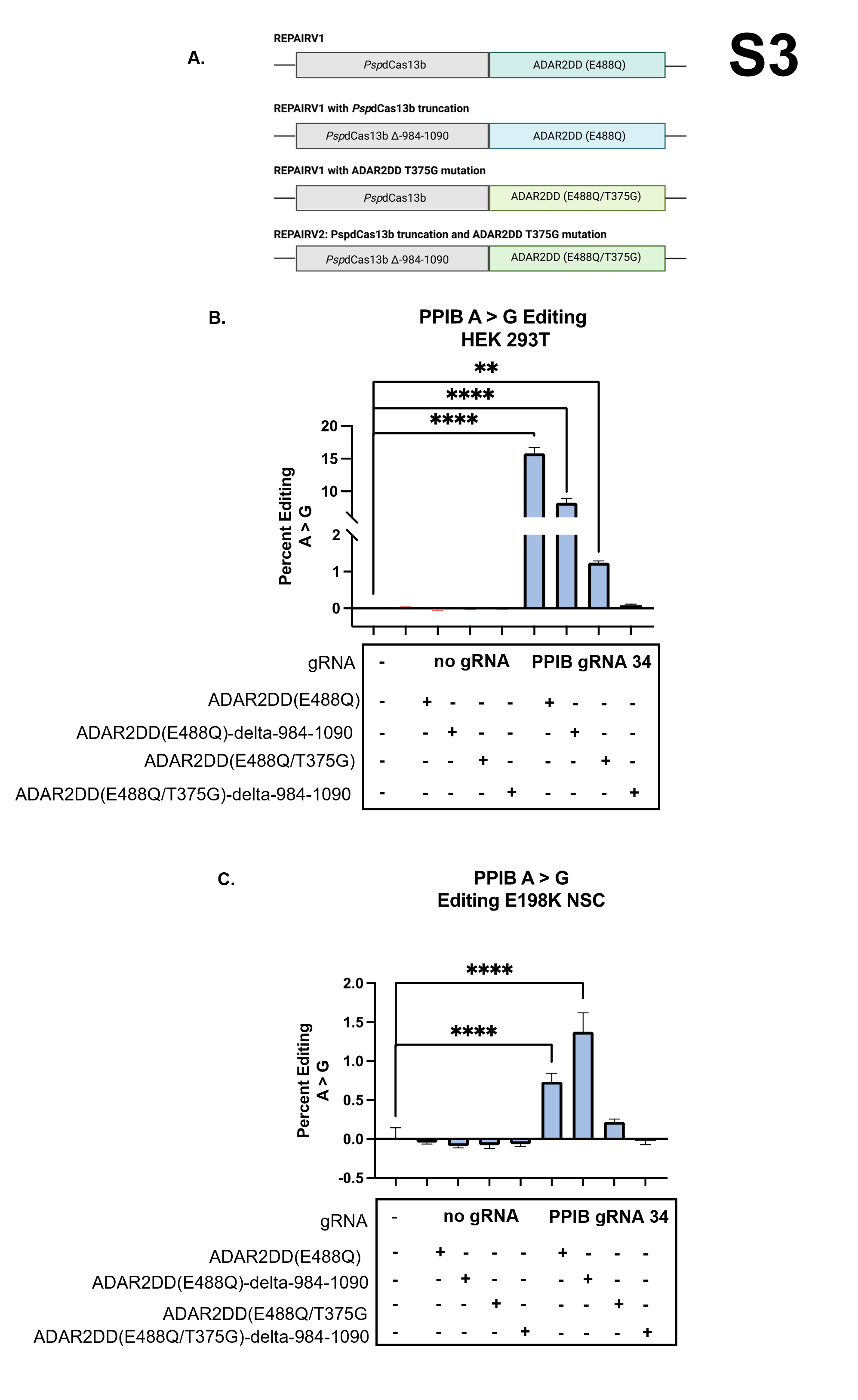

Supplement: sxae068_suppl_Supplementary_Material [file sxae068_suppl_supplementary_material.zip › Supplemental fig and Tables/SFig 3.png]

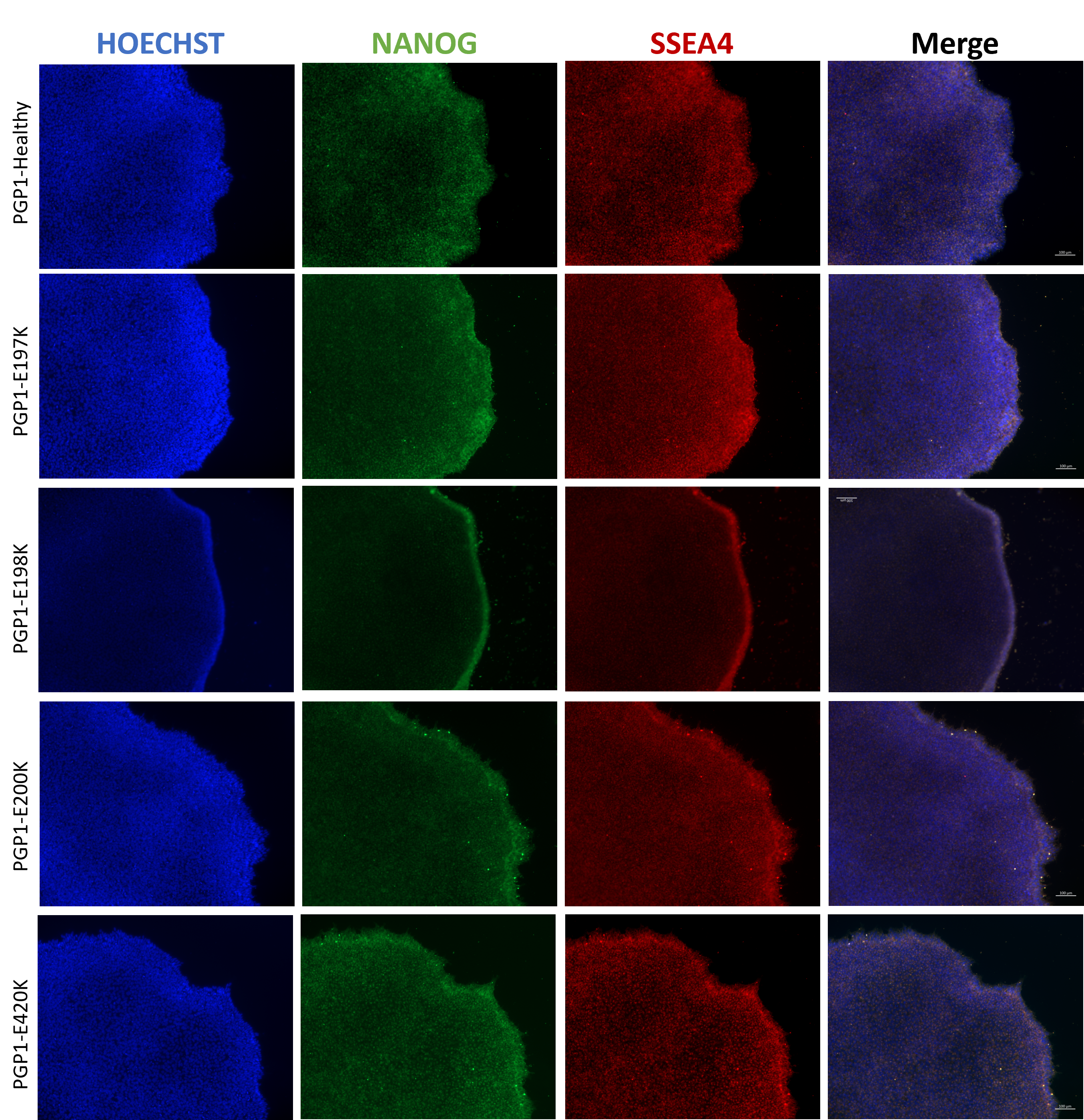

Supplement: sxae068_suppl_Supplementary_Material [file sxae068_suppl_supplementary_material.zip › Supplemental fig and Tables/SFig 4.png]
